# Whey protein lowers blood pressure and improves endothelial function and lipid biomarkers in adults with prehypertension and mild hypertension: results from the chronic Whey2Go randomized controlled trial^1^,^2^

**DOI:** 10.3945/ajcn.116.137919

**Published:** 2016-10-26

**Authors:** Ágnes A Fekete, Carlotta Giromini, Yianna Chatzidiakou, D Ian Givens, Julie A Lovegrove

**Affiliations:** 3 Hugh Sinclair Unit of Human Nutrition, Department of Food and Nutritional Sciences and Institute for Cardiovascular and Metabolic Research, School of Chemistry, Food and Pharmacy, and; 4 Food Production and Quality Research Division, School of Agriculture, Policy and Development, Faculty of Life Sciences, University of Reading, Reading, United Kingdom; and; 5 Department of Health, Animal Science and Food Safety, University of Milan, Milan, Italy

**Keywords:** blood pressure, cardiometabolic health, milk protein, vascular function, dairy, whey, caseinate, angiotensin-converting enzyme inhibition, flow-mediated dilatation, augmentation index

## Abstract

**Background:** Cardiovascular diseases (CVDs) are the greatest cause of death globally, and their reduction is a key public-health target. High blood pressure (BP) affects 1 in 3 people in the United Kingdom, and previous studies have shown that milk consumption is associated with lower BP.

**Objective:** We investigated whether intact milk proteins lower 24-h ambulatory blood pressure (AMBP) and other risk markers of CVD.

**Design:** The trial was a double-blinded, randomized, 3-way–crossover, controlled intervention study. Forty-two participants were randomly assigned to consume 2 × 28 g whey protein/d, 2 × 28 g Ca caseinate/d, or 2 × 27 g maltodextrin (control)/d for 8 wk separated by a 4-wk washout. The effects of these interventions were examined with the use of a linear mixed-model ANOVA.

**Results:** Thirty-eight participants completed the study. Significant reductions in 24-h BP [for systolic blood pressure (SBP): −3.9 mm Hg; for diastolic blood pressure (DBP): −2.5 mm Hg; *P* = 0.050 for both)] were observed after whey-protein consumption compared with control intake. After whey-protein supplementation compared with control intake, peripheral and central systolic pressures [−5.7 mm Hg (*P* = 0.007) and −5.4 mm Hg (*P* = 0.012), respectively] and mean pressures [−3.7 mm Hg (*P* = 0.025) and −4.0 mm Hg (*P* = 0.019), respectively] were also lowered. Flow-mediated dilation (FMD) increased significantly after both whey-protein and calcium-caseinate intakes compared with control intake [1.31% (*P* < 0.001) and 0.83% (*P* = 0.003), respectively]. Although both whey protein and calcium caseinate significantly lowered total cholesterol [−0.26 mmol/L (*P* = 0.013) and −0.20 mmol/L (*P* = 0.042), respectively], only whey protein decreased triacylglycerol (−0.23 mmol/L; *P* = 0.025) compared with the effect of the control. Soluble intercellular adhesion molecule 1 and soluble vascular cell adhesion molecule 1 were reduced after whey protein consumption (*P* = 0.011) and after calcium-caseinate consumption (*P* = 0.039), respectively, compared with after control intake.

**Conclusions:** The consumption of unhydrolyzed milk proteins (56 g/d) for 8 wk improved vascular reactivity, biomarkers of endothelial function, and lipid risk factors. Whey-protein supplementation also lowered 24-h ambulatory SBP and DBP. These results may have important implications for public health. This trial was registered at clinicaltrials.gov as NCT02090842.

## INTRODUCTION

Cardiovascular disease (CVD)^6^ remains the main cause of death in Western countries, although there has been a substantial decrease in its incidence in the past 2 decades. Despite the reduction in incidence, the prevalence of CVD is growing because of the increasing aging population in these countries (1). Of the modifiable risk factors of CVD, elevated blood pressure (BP) is a key risk factor. In the United Kingdom, nearly 30% of adults have hypertension, and a high percentage remain untreated (1). This lack of treatment can have serious consequences such as stroke, organ damage, or organ failure and death, which places an immense burden on the healthcare system. Note that even a small decrease in SBP of 2–5-mm Hg can translate into considerable reductions in CVD and total mortality (2). An improved quality of diet has been linked to a decrease in BP (3), and epidemiologic studies have shown an inverse association between milk consumption and BP (4). Milk is a complex food that contains several potential bioactive compounds; therefore, it remains to be confirmed which constituents may be responsible for the beneficial effect. Previous studies have linked milk-protein consumption with reduced BP (5); however, there has been an imbalance in the literature, because most attention has focused on the casein-derived lactotripeptides or other milk-protein hydrolysates (6) with a clear evidence gap on the effects of intact milk-protein isolates on BP (7). Milk proteins in liquid milk exist in intact forms, and evidence from robust randomized controlled trials (RCTs) would provide valuable information on the potential mechanism by which milk may affect BP. Furthermore, there is a clear need for a better assessment of BP, because the use of clinic BP may be confounded by the so-called white-coat effect. The 24-h ambulatory blood pressure (AMBP) is considered to be the gold standard, and AMBP predicts cardiovascular events, mortality, and morbidity that are associated with hypertension better than does clinic BP (8–10).

Vascular dysfunction is defined as the suppression of endothelium-dependent vasodilation or arterial stiffness (11, 12), and several factors such as hypertension, atherosclerosis, or diabetes can influence its rate of progression (13). Little attention has focused on the potential impact of dairy proteins on vascular function. Pal and Ellis (7) reported a decrease in BP after both whey-protein and sodium-caseinate supplementation for 12 wk; however, they showed improvements in arterial stiffness only after whey-protein consumption. This result may indicate that whey protein has an additional beneficial effect on the cardiovascular system compared with that of caseinate; however, this possibility remains to be confirmed.

Therefore, our hypothesis was that whey-protein and calcium-caseinate supplementation, compared with control intake, would reduce 24-h AMBP, improve vascular function, and beneficially affect other cardiometabolic risk markers. This hypothesis was tested in a randomized, controlled, double-blinded, 3-way–crossover dietary intervention in prehypertensive and mildly hypertensive men and women.

## METHODS

### Subjects

Healthy, nonsmoking men and women with mildly elevated BP of 120/80–159/99 mm Hg who were aged 30–77 y and were not taking antihypertensive or cholesterol-lowering medications were recruited for this study through database, local newspaper, and electronic advertisements. This study was conducted at the Hugh Sinclair Unit of Human Nutrition, University of Reading, between February 2014 and February 2015. Inclusion criteria were as follows: a signed consent form; BP from 120/80 to 159/99 mm Hg; aged 30–77 y; BMI (in kg/m^2^) from 20 to 40; glucose concentration <7 mmol/L (not diagnosed with diabetes); cholesterol concentration <8 mmol/L; triacylglycerol concentration <4 mmol/L; normal liver and kidney function; and hemoglobin concentration >110 and >140 g/dL for women and men, respectively. Participants were excluded on the basis of the following criteria: a milk allergy or lactose intolerance; celiac disease or renal, gastrointestinal, respiratory, endocrine, or liver disease or cancer; surgery in the previous 6 mo; secondary hypertension; excess alcohol consumption (drinking >280 mL and >210 mL ethanol/wk for men and women, respectively); smoking; being a vegan; taking a nutritional supplement (e.g., fish oil, protein shakes, or vitamins); anemia; and pregnancy, lactation, or planning a pregnancy. Blood donation was not allowed for 3 mo before or during the study. All volunteers provided a signed consent form at the screening visit before their inclusion in the study and agreed to adhere to the trial guidelines and give notification of any noncompliance. To detect the arithmetic mean ± SEM SBP intergroup difference (primary outcome) of 4.9 ± 6.5 mm Hg (7, 14) with 90% power and at a 5% significance level, 37 participants were required, which increased to 42 participant to account for a 12% dropout rate (*n* = 5).

### Study design

The study was approved by the Research Ethics Committee at the University of Reading (Research Ethics Committee project 12/40), and the protocol was conducted in accordance with the ethical standards laid down in the 1964 Declaration of Helsinki. This trial was registered at clinicaltrials.gov as NCT02090842. The study was a randomized, double-blinded, controlled, 3-way–crossover, dietary intervention study with a 2-wk run-in period before the beginning of the trial. There were two 4-wk washout periods that separated the 3 intervention arms of 8 wk. The total duration of the study was 32 wk. The study design is shown in **Supplemental Figure 1**. The volunteers were randomly assigned by an independent researcher with the use of an Excel-based random-assignment program (ExtendOffice 12.5) (15) who was also responsible for the treatment allocation. Equal numbers of volunteers were allocated within 6 treatment sequences (ABC, ACB, BAC, BCA, CAB, and CBA). A double-blinded protocol was maintained throughout the study, and during the statistical analysis. The codes for the study drinks were kept under lock at Volac International Ltd. (Cambridge) and the University of Reading.

The primary outcome was 24-h AMBP, and secondary outcomes were vascular reactivity measured with the use of FMD and changes in plasma lipids, markers of insulin resistance, inflammatory markers, and arterial stiffness measured with the use of a pulse wave analysis and digital volume pulse (DVP).

### Study drinks

The 3 treatment products were 90% whey-protein isolate (Volac International Ltd.), 90% calcium caseinate (Garret Ingredients), and maltodextrin (control) (MyProtein) and were packed into identical foil sachets that were labeled with study codes. All products were commercially available. Each protein-containing sachet contained 28 g powder and 27 g maltodextrin in the control sachets, which provided closely matched amounts of energy (1596 kJ/100 g for whey protein, 1519 kJ/100 g for calcium caseinate, and 1628 kJ/100 g for maltodextrin) (for nutrient compositions, see **Supplemental Table 1**). Calcium caseinate was used in this study instead of sodium caseinate to reduce the potential bias toward the effect of whey protein because sodium may unfavorably affect BP. Volunteers were asked to consume 2 sachets/d mixed with 250 mL H_2_O. Participants were also given a choice of sugar-free flavor concentrates [vanilla, banana, tropical fruit, or chocolate (MyProtein)] that were to be added before consumption to mask any possible taste of the supplements and to increase compliance. Furthermore, volunteers were informed at the start of the study that all 3 treatments were substantially different from one another in terms of taste and appearance to reduce the possibility of them identifying the protein interventions. Subjects were required to refrain from mixing the sachet content with milk to avoid increasing their milk intake. Participants were also asked to maintain isoenergetic diets to account for the extra energy intake from the consumption of the study drinks. Volunteers were provided with individual nutritional advice by a dietitian and a nutritionist on specific strategies for the incorporation of study drinks into their diets without a substantial change to their habitual food intakes. Participants recorded the exchanged foods (types and weights) on tailored diary sheets, which were evaluated at each study visit, and feedback was provided to the volunteers as to their compliance to the interventions. Subjects were also required to record their consumption of the sachets by marking tick boxes as well as by keeping empty sachets to monitor compliance.

### Study visits

At the screening visit, data on height and weight were collected. Height was measured to the nearest 0.5 cm with the use of a wall-mounted stadiometer. Furthermore, weight and BMI were measured with the use of a regularly calibrated bioelectroimpedence scale (Tanita BC-418 digital scale; Tanita Europe) with the use of standard settings (normal body type and +1 kg for clothing) while wearing light clothing. A fasted serum blood sample was collected to assess serum glucose, total cholesterol, triacylglycerol, creatinine, bilirubin, uric acid, alanine aminotransferase, γ-glutamyl transpeptidase, and alkaline phosphatase with the use of an autoanalyzer [ILAB600; Werfen (UK) Ltd.]. Participants were also checked for anemia by a full blood count. After 20 min rest, BP measurement was taken on the upper left arm with the use of an automatic BP monitor (A/A-grade automated oscillometric BP monitors; A&D Instruments Ltd.), which recorded BP readings every 5 min up to 30 mins in a temperature-controlled, quiet environment. The mean of 6 readings was used to assess resting BP to the nearest 0.5 mm Hg. Participants were required to complete a food-frequency questionnaire (FFQ) (European Prospective Investigation into Cancer and Nutrition-Norfolk FFQ v.6) and were asked to remain seated and to refrain from speaking. FFQs were subsequently analyzed with the use of FETA (EPIC, Norfolk) for Windows GLP v2.53 software (16).

The run-in period started with a familiarization visit (visit 0) in the clinical room where the study visits were conducted at the Hugh Sinclair Unit of Human Nutrition to familiarize participants with the environment and, thus, decrease the so-called white-coat effect on the clinic BP and vascular measurements. All volunteers had a one-by-one consultation with the study researcher. During this session, volunteers were provided with verbal and written instructions on the consumption and recording of the consumption of the study drinks, the maintenance of an isocaloric diet, the use of the 24-h AMBP, and the recording of 4-d estimated dietary intake (including 3 weekdays and 1 weekend day). Participants were required to complete the diaries on 6 occasions before each study visit. Portion sizes were estimated with the use of household measures or portion-size images (17), which were later quantified by a trained dietitian with the use of food-portion tables (18). Dietplan 6.6 software (Forestfield Software) was used to assess nutrient intakes of participants for which the National Diet and Nutrition Survey nutrient databank was implemented (19). Volunteers were specifically asked their body weight and reminded at each study visit to maintain their body weights and physical activities and to continue any permitted medication use without any change in the dose during the interventions of which they had to keep record. All vascular measurement techniques and their implementation were clearly explained and shown to the participants.

At baselines (visits 1, 3, and 5) and at the end of all 3 intervention periods (visit 2, 4, and 6), overnight (12-h) fasting venous blood samples were collected for biochemical analysis. Participants were required to refrain from alcohol and aerobic exercises for 24-h before visits, and they had to orally confirm that they adhered to this protocol. Volunteers consumed a low-fat and low-protein meal the evening before each visit and drank only low-nitrate water (Buxton Mineral Water; Nestlé Waters UK). Body weight and body composition were measured as specified previously upon arrival to the nutrition unit. Hand-grip strength was assessed with the use of a hand-grip dynamometer (SU424; JP Lennard Ltd.). Volunteers were asked to perform 3 maximum hand-grip tests with each hand in a standing position with their arms along their bodies. The mean of the 3 measurements calculated the hand-grip strength. Participants rested for 30 min in a supine position in a quiet, temperature-controlled room (20°C ± 1°C) before vascular testing. Before the commencement of vascular measurements, clinic BP was taken on the left upper arm with the use of a validated BP monitor (Omron MX2 Digital Automatic Upper Arm Blood Pressure Monitor; OMRON), and the mean of 3 consecutive measurements was recorded. All measurements were conducted by the same researcher (except for the DVP, which was operated by multiple trained researchers) and at the same time of day for all visits. Premenopausal women attended all visits at the same phase of their menstrual cycles.

### Twenty-four-hour AMBP and assessments of vascular function

AMBP was measured with the use of A/A-grade automated oscillometric AMBP monitors (A&D Instruments Ltd.). Twenty-four-hour AMBP and heart rate were recorded every 30 min from 0700 to 2159 and every 60 min from 2200 to 0659 no later than 48 h before clinical visits. Mean 24-h day and night measurements were assessed with the use of sleep times that were recorded on the volunteer activity forms. Pulse pressure was expressed as the difference between SBP and DBP.

FMD was performed by a single, trained researcher as previously described (20). The technique assessed the endothelial-dependent vasodilation of the vasculature with the use of a CX50 Ultrasound System (Philips) according to standard guidelines (21). Electrocardiogram-gated image acquisition was performed at 0.25 frames/s with the use of image-grabbing software (Medical Imaging Applications LLC). The analysis of the obtained images was conducted by a single researcher with the use of wall-tracking software (Vascular Research Tools 5; Medical Imaging Applications LLC). FMD was calculated as the maximum change in the postocclusion brachial artery diameter, which was expressed as the percentage of the baseline diameter [flow-medated dilatation percentage (%FMD)]. The %FMD was assessed 3 times on each image, and the mean was calculated. The arterial stiffness of peripheral vessels was assessed in triplicate as detailed elsewhere (22) with the use of a radial pulse-wave analysis (SphygmoCor; AtCor Medical). The pulse-wave analysis determined the augmentation index, which was corrected for a heart rate of 75 beats/min (percentage). The digital volume pulse (Pulse Trace PCA2; Micro Medical Ltd.) provided an indication of the stiffness index (meters per second) and reflection index (percentage) as measures of arterial stiffness and vascular tone, respectively (20).

### Biochemical analyses

Fasted blood samples were centrifuged at 1800 × *g* for 15 min at 20°C (serum separator blood collection tube; VACUETTE, Greiner Bio-One) to obtain serum and at 1800 × *g* for 10 min at 4°C (blood collection tubes containing lithium heparin and EDTA; VACUETTE; Greiner Bio-One) to obtain plasma. Samples were kept at −20°C. Lipids (total cholesterol, HDL cholesterol, and triacylglycerol), glucose, nonesterified fatty acid, and C-reactive protein were quantified from serum with the use of an autoanalyzer [ILAB600; Werfen (UK) Ltd.; reagents and analyzer: Instrumentation Laboratory Ltd.; nonesterified fatty acid reagent: Alpha Laboratories). LDL cholesterol was estimated with the use of Friedewald’s formula (23). Insulin resistance was determined with the use of HOMA-IR, and insulin sensitivity was assessed with the original and revised quantitative insulin sensitivity check indexes with the use of standard equations (24). ELISA kits were used to determine serum insulin (Dako Ltd.), intercellular and vascular adhesion molecules, E-selectin, and P-selectin; high-sensitivity kits were used to determine IL-6 and TNF-α (R&D Systems Europe Ltd.). Plasma nitrite and nitrate were measured with the use of HPLC with online reduction of nitrate to nitrite and postcolumn derivatization with the Griess reagent (ENO-30 Analyzer; Eicom) as described elsewhere (25). Serum angiotensin-converting enzyme (ACE) activity was determined by a fluorometric assay as described elsewhere (26). Mean intra-assay and interassay CVs were <5% for the automated assays and <10% for other assays.

### In vitro ACE inhibitory effects of intervention products

To assess the ACE inhibitory (ACEi) effect of intervention proteins and the control, the study products were digested in vitro on the basis of the method of Mills et al. (27) with minor modifications. The total digesta were filtered with the use of-3kDa VIVASPIN 20 centrifuge tubes (Sartorius AG) to mimic in vivo intestinal absorption. The ACEi effect of intervention proteins and the control were measured with the use of a spectrophotometric assay with the substrate furanacryloyl-Phe-Gly-Gly and ACE, which were extracted from rabbit lung (both from Sigma-Aldrich) (28, 29). The ACEi effect was expressed as
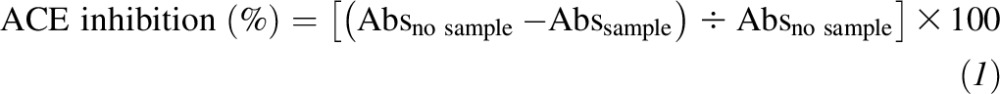
Abs_no sample_ is the absorbance of the enzyme-substrate mixture in the absence of digested protein, and Abs_sample_ is the absorbance of the enzyme-substrate mixture in the presence of digested protein.

### Statistical analyses

Statistical analyses were performed with the use of SAS version 9.4 software (SAS Institute Inc.). Suitable checks for normality were performed (evaluated with the use of histograms and quantile-quantile plots) with IBM SPSS Statistics version 21 software (SPSS Inc.), and data were logarithmically transformed as appropriate. The data from participants who withdrew from the study were excluded in the analysis because a per-protocol analysis was used. The effects of treatments were evaluated by implementing a linear mixed-model ANOVA with the use of the difference from baseline (Δ visit 2 − visit 1) as a dependent variable and baseline values of the variable of interest, BMI, age, sex, and intervention treatment as prognostic variables. *P* ≤ 0.05 was considered significant. Data presented in the text, tables, and figures represent arithmetic means ± SEMs.

## RESULTS

### Study participation and compliance

**FIGURE 1:**
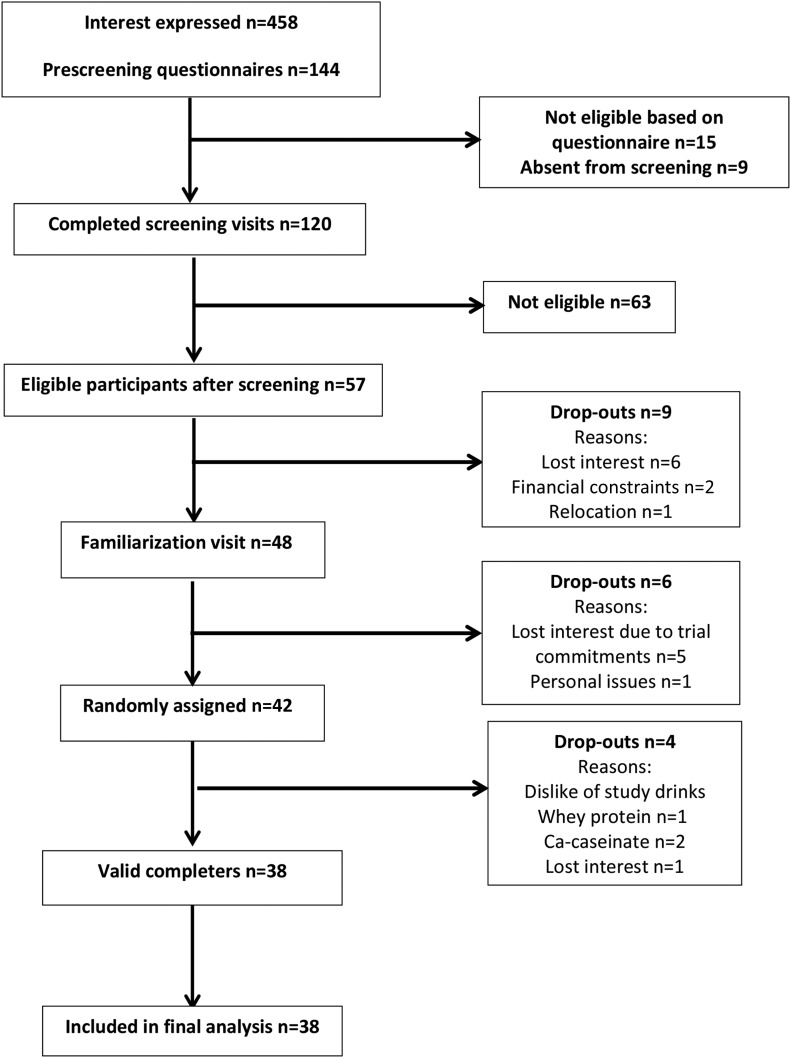
Participant inclusion flowchart of the Whey2Go study.

A total of 42 volunteers were randomly assigned to interventions, of whom 38 completed the study. Four participants withdrew during the study [2 subjects dropped out because of a dislike of calcium caseinate, 1 subject dropped out because of a dislike of whey protein, and 1 subject dropped out because of a loss of interest (see **Figure 1** for flowchart)]. **Table 1** summarizes the baseline characteristics of completing participants. On the basis of sachet counting, compliance was 91.0% ± 1.7%, 88.6% ± 2.1%, and 91.8% ± 1.4% for whey protein, calcium-caseinate, and control consumption, respectively (*n* = 38); and on the basis of tick-sheet recordings, compliance was 91.7% ± 1.9%, 89.9% ± 2.2%, and 93.6% ± 1.8% for whey protein, calcium-caseinate, and control consumption, respectively (*n* = 37; 1 volunteer failed to complete the form). There were no significant differences between intervention proteins and the control in terms of compliance assessed both with the use of a sachet count (overall treatment effect: *P* = 0.400) and tick sheet (overall treatment effect: *P* = 0.545).

**TABLE 1:** Baseline characteristics of participants^1^

	Value
Total (men/women), *n*	38 (20/18)
Age, y	52.9 ± 2.1^2^
BMI, kg/m^2^	27.1 ± 0.8
SBP, mm Hg	135.5 ± 2.2
DBP, mm Hg	84.0 ± 1.9
Total cholesterol, mmol/L	5.11 ± 0.2
Triacylglycerol, mmol/L	1.12 ± 0.1
Glucose, mmol/L	5.51 ± 0.1

1 DBP, diastolic blood pressure; SBP, systolic blood pressure.

2 Mean ± SEM (all such values).

### Anthropometric measurements

The body composition of participants remained stable during interventions, and there were no significant treatment effects on those variables (for details, see **Supplemental Table 2**).

### Twenty-four-hour AMBP

For the primary outcome 24-h AMBP, there were significant overall treatment effects (*P* = 0.027) (**Table 2**). Whey-protein consumption reduced 24-h SBP to a greater extent than did the control and also reduced daytime SBP compared with the effect of calcium caseinate (*P* = 0.048), and there was a tendency for lower daytime SBP after whey-protein supplementation than after control intake (*P* = 0.053). Similarly, whey protein lowered 24-h DBP compared with the effect of the control (*P* = 0.050) and lowered daytime DBP compared with the effect of calcium caseinate (*P* = 0.035), and there was a tendency for lower daytime DBP compared with the effect of the control (*P* = 0.077). No treatment effect was seen for nighttime SBP, DBP, pulse pressure, or heart rate. However, there was a significant treatment effect for clinic SBP, whereby whey protein decreased SBP compared with the effect of maltodextrin (Table 2; for heart-rate and pulse-pressure results, see **Supplemental Table 3**).

**TABLE 2:** Twenty-four-hour ambulatory and clinic blood pressure at baseline (week 0) and postintervention (week 8) for whey protein, calcium-caseinate, and control interventions^1^

	Whey protein			Calcium caseinate			Control			
	Baseline	Post	Δ	Baseline	Post	Δ	Baseline	Post	Δ	*P*^2^
24-h SBP, mm Hg	130.0 ± 2.2	127.1 ± 2.3	−2.9 ± 1.1^a^	129.7 ± 2.0	130.3 ± 2.6	0.6 ± 1.7^a,b^	129.5 ± 2.3	130.5 ± 2.3	1.0 ± 1.1^b^	0.041
Day SBP, mm Hg	134.2 ± 2.2	131.3 ± 2.4	−2.8 ± 1.2^a^	133.7 ± 2.2	135.4 ± 2.7	1.7 ± 1.7^b^	133.4 ± 2.2	134.8 ± 2.4	1.3 ± 1.2^a,b^	0.027
Night SBP, mm Hg	115.2 ± 2.4	112.2 ± 2.5	−3.3 ± 1.5	115.5 ± 2.4	113.6 ± 3.0	−2.0 ± 2.5	114.7 ± 2.3	115.7 ± 2.4	0.9 ± 1.4	0.110
24-h DBP, mm Hg	78.9 ± 1.5	76.9 ± 1.5	−2.0 ± 0.7^a^	78.6 ± 1.3	79.0 ± 1.6	0.3 ± 1.0^a,b^	78.0 ± 1.5	78.4 ± 1.6	0.5 ± 0.6^b^	0.039
Day DBP, mm Hg	81.9 ± 1.6	79.8 ± 1.6	−2.1 ± 0.8^a^	81.6 ± 1.5	82.8 ± 1.7	1.2 ± 1.1^b^	80.8 ± 1.5	81.5 ± 1.6	0.7 ± 0.7^a,b^	0.027
Night DBP, mm Hg	68.0 ± 1.5	66.2 ± 1.5	−1.8 ± 0.8	67.4 ± 1.6	66.1 ± 1.8	−1.3 ± 1.5	67.2 ± 1.5	67.7 ± 1.5	0.5 ± 1.0	0.086
Clinic SBP, mm Hg	132.1 ± 2.1	127.9 ± 2.0	−4.2 ± 1.3^a^	129.3 ± 2.0	128.4 ± 2.0	−0.9 ± 1.5^a,b^	130.2 ± 2.2	130.9 ± 2.2	0.7 ± 1.4^b^	0.035
Clinic DBP, mm Hg	76.7 ± 1.6	74.6 ± 1.5	−2.1 ± 0.8	74.9 ± 1.6	73.6 ± 1.7	−1.3 ± 0.8	74.8 ± 1.5	75.2 ± 1.8	0.5 ± 0.9	0.100
Clinic PP, mm Hg	55.5 ± 1.6	53.3 ± 1.3	−2.2 ± 1.1	54.4 ± 1.9	54.8 ± 1.3	0.4 ± 1.1	55.4 ± 1.6	55.7 ± 1.5	0.2 ± 1.0	0.061

1 All values are means ± SEMs. *n* = 38. Baselines were significantly different (*P* ≤ 0.05) from one another except for 24-h SBP and DBP and day DBP. Different superscript letters within a row refer to treatment groups different from one another (*P* ≤ 0.05). DBP, diastolic blood pressure; PP, pulse pressure; SBP, systolic blood pressure; Δ change from baseline.

2 Overall between-group treatment effects for each Δ were obtained with the use of a linear mixed-model ANOVA with baseline values for the variable of interest and prognostic values such as age, sex, and BMI. Tukey-Kramer correction was used for the post hoc analysis to adjust for multiple testing.

### Vascular function

There was a significant treatment effect on the %FMD whereby both whey protein and calcium caseinate increased the %FMD compared with the effect of the control. The augmentation index, which is a measure of arterial stiffness, was not affected by the study drinks; however, compared with the control, whey protein reduced both peripheral and central systolic and mean pressures. Other measures of arterial stiffness that were assessed with the use of the DVP were not different between treatment groups (**Table 3**).

**TABLE 3:** Vascular measurements at baseline (week 0) and postintervention (week 8) for whey protein, calcium-caseinate, and control interventions^1^

	Whey protein			Calcium caseinate			Control			
	Baseline	Post	Δ	Baseline	Post	Δ	Baseline	Post	Δ	*P*^2^
Endothelial function										
%FMD	4.79 ± 0.3	5.38 ± 0.4	0.59 ± 0.2^a^	4.83 ± 0.3	4.94 ± 0.3	0.11 ± 0.2^a^	4.79 ± 0.3	4.07 ± 0.3	−0.72 ± 0.2^b^	<0.001
Arterial stiffness										
Assessed by PWA										
Augmentation index at 75 beats/min, %	17.6 ± 2.2	17.0 ± 2.2	−0.6 ± 0.8	18.2 ± 2.3	17.1 ± 2.4	−1.0 ± 0.8	18.2 ± 2.3	17.2 ± 2.1	−1.0 ± 0.8	0.946
Peripheral SP, mm Hg	132.3 ± 2.2	127.5 ± 2.0	−4.8 ± 1.4^a^	129.1 ± 2.0	129.0 ± 2.0	−0.1 ± 1.4^a,b^	130.1 ± 2.2	131.0 ± 2.2	0.9 ± 1.4^b^	0.007
Peripheral DP, mm Hg	76.6 ± 1.5	74.7 ± 1.5	−1.9 ± 0.8	74.7 ± 1.6	73.7 ± 1.6	−1.0 ± 0.8	74.7 ± 1.5	75.3 ± 1.8	0.6 ± 0.9	0.096
Peripheral MP, mm Hg	95.9 ± 2.0	92.5 ± 2.0	−3.4 ± 0.9^a^	94.1 ± 1.8	92.9 ± 1.9	−1.2 ± 1.0^a,b^	94.3 ± 1.8	94.6 ± 2.0	0.3 ± 0.9^b^	0.032
Central SP, mm Hg	121.7 ± 2.5	116.7 ± 2.3	−5.0 ± 1.3^a^	118.6 ± 2.3	117.9 ± 2.3	−0.7 ± 1.3^a,b^	119.2 ± 2.4	119.6 ± 2.6	0.4 ± 1.3^b^	0.011
Central DP, mm Hg	77.7 ± 1.6	75.6 ± 1.5	−2.1 ± 0.8	75.8 ± 1.7	74.8 ± 1.7	−1.0 ± 0.8	75.8 ± 1.5	76.4 ± 1.8	0.6 ± 0.9	0.094
Central MP, mm Hg	96.6 ± 1.8	93.3 ± 1.7	−3.3 ± 0.8^a^	94.1 ± 1.8	93.4 ± 1.8	−0.7 ± 0.9^a,b^	94.2 ± 1.8	94.9 ± 2.0	0.7 ± 1.0^b^	0.023
Assessed by DVP										
Stiffness index, m/s	7.53 ± 0.4	7.42 ± 0.4	−0.11 ± 0.3	8.01 ± 0.4	7.92 ± 0.4	−0.09 ± 0.2	7.59 ± 0.4	7.55 ± 0.4	−0.04 ± 0.3	0.542
Reflection index, %	62.1 ± 2.9	66.2 ± 2.6	4.1 ± 2.3	65.0 ± 2.3	65.3 ± 2.7	0.3 ± 1.9	64.7 ± 2.4	64.7 ± 2.5	0.01 ± 1.6	0.515
PPT, m/s	246.5 ± 10.0	249.1 ± 9.9	2.6 ± 9.3	233.1 ± 10.5	235.2 ± 10.0	2.1 ± 5.2	244.2 ± 10.4	242.9 ± 9.5	−1.3 ± 8.6	0.463

1 All values are means ± SEMs. *n* = 38 (except for FMD and DVP, for which *n* = 36). Baselines were significantly different (*P* ≤ 0.05) from one another except for peripheral DP and MP and central DP. Different superscript letters within a row refer to treatment groups differing from one another, *P* ≤ 0.05. DP, diastolic pressure; DVP, digital volume pulse; FMD; flow-mediated dilation; MP, mean pressure; PPT, peak to peak; PWA, pulse-wave analysis; SP, systolic pressure; Δ change from baseline; %FMD, flow-mediated dilatation percentage.

2 Overall between-group treatment effects for each Δ were obtained with the use of linear mixed-model ANOVA with baseline values for the variable of interest and prognostic values such as age, sex, BMI. Tukey-Kramer correction was used for the post hoc analysis to adjust for multiple testing.

### Blood analyses

There was a significant reduction in total cholesterol after whey protein and calcium-caseinate consumption than after control intake. Triacylglycerol also decreased significantly in the whey-protein group compared with in the control group and almost reached significance (*P* = 0.055) between calcium caseinate and the control. No difference was seen on any other components of the lipid profile or indexes of insulin resistance and sensitivity. Although whey protein, compared with the control, lowered soluble intercellular adhesion molecule-1 (sICAM-1) significantly, calcium caseinate reduced soluble vascular cell adhesion molecule 1 (sVCAM-1). The protein supplements had no effects on any inflammatory markers or on plasma nitric oxide and serum ACE activity (**Table 4**).

**TABLE 4:** Fasting lipid profile, indexes of insulin resistance, and vascular biomarkers at baseline (week 0) and postintervention (week 8) for whey protein, calcium-caseinate, and control interventions^1^

	Whey protein			Calcium caseinate			Control			
	Baseline	Post	Δ	Baseline	Post	Δ	Baseline	Post	Δ	*P*^2^
Fasting lipid profile										
Total cholesterol, mmol/L	5.05 ± 0.2	4.88 ± 0.1	−0.18 ± 0.1^a^	5.01 ± 0.2	4.89 ± 0.2	−0.12 ± 0.1^a^	5.05 ± 0.2	5.12 ± 0.2	0.08 ± 0.1^b^	0.010
LDL cholesterol, mmol/L	3.09 ± 0.1	3.00 ± 0.1	−0.09 ± 0.1	3.08 ± 0.1	3.05 ± 0.1	−0.03 ± 0.1	3.13 ± 0.1	3.15 ± 0.1	0.01 ± 0.1	0.337
HDL cholesterol, mmol/L	1.36 ± 0.1	1.34 ± 0.1	−0.02 ± 0.02	1.35 ± 0.1	1.31 ± 0.1	−0.04 ± 0.03	1.34 ± 0.1	1.37 ± 0.1	0.02 ± 0.01	0.160
Total cholesterol:HDL cholesterol, mmol/L	4.00 ± 0.2	3.81 ± 0.2	−0.19 ± 0.1	3.97 ± 0.2	3.95 ± 0.2	−0.01 ± 0.1	4.02 ± 0.2	4.02 ± 0.2	0.00 ± 0.1	0.094
LDL cholesterol:HDL cholesterol, mmol/L	2.48 ± 0.2	2.38 ± 0.1	−0.10 ± 0.1	2.47 ± 0.2	2.50 ± 0.2	0.03 ± 0.1	2.53 ± 0.2	2.49 ± 0.2	−0.04 ± 0.1	0.168
Triacylglycerol, mmol/L	1.31 ± 0.1	1.16 ± 0.1	−0.15 ± 0.0^a^	1.28 ± 0.1	1.16 ± 0.1	−0.12 ± 0.1^a,b^	1.25 ± 0.1	1.34 ± 0.1	0.08 ± 0.1^b^	0.018
Indexes of insulin resistance										
Glucose, mmol/L	5.04 ± 0.1	5.02 ± 0.1	−0.03 ± 0.01	5.03 ± 0.1	5.10 ± 0.1	0.07 ± 0.1	5.00 ± 0.1	5.12 ± 0.1	0.12 ± 0.1	0.111
Insulin, pmol/L	52.4 ± 5.3	51.4 ± 5.0	−1.17 ± 4.1	46.1 ± 4.3	51.7 ± 4.2	5.53 ± 2.7	47.6 ± 4.8	52.8 ± 5.0	5.25 ± 3.2	0.498
NEFAs, μmol/L	487.7 ± 35.0	414.4 ± 29.1	−73.3 ± 30.7	528.7 ± 34.1	408.6 ± 28.4	−120.1 ± 22.0	533.1 ± 32.8	464.0 ± 29.2	−69.1 ± 25.8	0.091
HOMA-IR	1.99 ± 0.2	1.94 ± 0.2	−0.05 ± 0.1	1.75 ± 0.2	1.98 ± 0.2	0.23 ± 0.1	1.79 ± 0.2	2.03 ± 0.2	0.24 ± 0.1	0.378
QUICKI	0.501 ± 0.0	0.502 ± 0.0	0.001 ± 0.0	0.503 ± 0.0	0.500 ± 0.0	−0.003 ± 0.0	0.503 ± 0.0	0.500 ± 0.0	−0.003 ± 0.0	0.135
rQUICKI	0.510 ± 0.0	0.511 ± 0.0	0.001 ± 0.0	0.511 ± 0.0	0.509 ± 0.0	−0.002 ± 0.0	0.512 ± 0.0	0.509 ± 0.0	−0.003 ± 0.0	0.102
Vascular biomarkers										
sVCAM-1, ng/mL	637.3 ± 21.0	639.6 ± 22.5	2.3 ± 11.1^a,b^	641.6 ± 22.5	618.5 ± 21.4	−23.1 ± 11.7^a^	627.7 ± 20.3	649.4 ± 23.2	21.7 ± 9.9^b^	0.011
sICAM-1, ng/mL	224.7 ± 10.6	213 ± 9.0	−11.7 ± 5.0^a^	221.7 ± 10.4	218.4 ± 9.0	−3.2 ± 5.6^a,b^	223.6 ± 8.9	228.9 ± 10.7	5.3 ± 4.9^b^	0.039
E-selectin, ng/mL	33.7 ± 2.1	34.15 ± 2.0	0.45 ± 0.8	34.46 ± 2.2	34.90 ± 2.1	0.44 ± 0.7	34.43 ± 2.2	36.32 ± 2.4	1.89 ± 0.8	0.303
P-selectin, ng/mL	33.5 ± 0.4	33.6 ± 0.3	0.09 ± 0.1	33.9 ± 0.3	33.5 ± 0.4	−0.41 ± 0.2	33.9 ± 0.3	33.6 ± 0.4	−0.28 ± 0.2	0.143
CRP, mg/L	1.25 ± 0.2	1.14 ± 0.2	−0.11 ± 0.2	1.13 ± 0.1	1.46 ± 0.3	0.33 ± 0.3	1.10 ± 0.2	1.28 ± 0.2	0.18 ± 0.2	0.906
IL-6, pg/mL	1.31 ± 0.1	1.20 ± 0.1	−0.11 ± 0.1	1.28 ± 0.1	1.27 ± 0.1	−0.02 ± 0.1	1.21 ± 0.1	1.25 ± 0.1	0.04 ± 0.1	0.926
TNF-α, pg/mL	1.23 ± 0.1	1.24 ± 0.1	0.01 ± 0.1	1.24 ± 0.1	1.25 ± 0.1	0.01 ± 0.1	1.22 ± 0.1	1.27 ± 0.1	0.05 ± 0.1	0.856
NO_2_, μmol/L	1.45 ± 0.1	1.51 ± 0.1	0.06 ± 0.2	1.41 ± 0.1	1.49 ± 0.1	0.08 ± 0.2	1.51 ± 0.1	1.35 ± 0.1	−0.17 ± 0.1	0.842
NO_3_, μmol/L	12.8 ± 1.9	12.1 ± 1.6	−0.75 ± 1.4	12.0 ± 1.2	10.7 ± 1.1	−1.30 ± 1.1	10.8 ± 1.1	11.7 ± 1.7	−0.98 ± 1.2	0.811
ACE activity, U/L	4.65 ± 0.2	4.83 ± 0.2	0.18 ± 0.1	4.76 ± 0.2	5.01 ± 0.2	0.25 ± 0.1	4.58 ± 0.2	5.03 ± 0.2	0.45 ± 0.1	0.503

1 All values are means ± SEMs. *n* = 38 (except for sICAM-1 and E-selectin, for which *n* = 36; for P-selectin and IL-6, for which *n* = 37; and for CRP, for which *n* = 35). Baselines were not significantly different (*P* ≤ 0.05) from one another except for E-selectin and IL-6. Data were log transformed for CRP, IL-6, and TNF-α. Different superscript letters within a row refer to treatment groups different from one another (*P* ≤ 0.05). ACE, angiotensin-converting enzyme; CRP, C-reactive protein; NEFA, nonesterified fatty acid; QUICKI, quantitative insulin sensitivity index, rQUICKI, revised quantitative insulin sensitivity index; sICAM-1, soluble intercellular adhesion molecule 1; sVCAM-1, soluble vascular cell adhesion molecule 1; Δ, change from baseline.

2 Overall between-group treatment effects for each Δ were obtained with the use of a linear mixed-model ANOVA with baseline values for the variable of interest and prognostic values such as age, sex, and BMI. Tukey-Kramer correction was used for the post hoc analysis to adjust for multiple testing.

### Diet

No overall treatment effect was observed for nutrient intake apart from that for protein (higher for the milk-protein groups than for the control group), carbohydrate (higher for the control than for the whey protein and casein), and calcium intake (higher for the calcium-caseinate group than for the whey-protein and control groups) (**Table 5**). On the basis of the FFQ, the mean ± SEM milk intake of participants was 312 ± 33 mL/d; consumption of yogurt, fromage frais, and other dairy desserts was 74 ± 13 mL/d; cheese intake was 19.3 ± 3.2 g/d; and butter consumption was 2.8 ± 0.6 g/d.

**TABLE 5:** Reported nutrient intake at baseline (week 0) and postintervention (week 8) for whey protein, calcium-caseinate and control interventions^1^

	Whey protein			Calcium caseinate			Control			
	Baseline	Post	Δ	Baseline	Post	Δ	Baseline	Post	Δ	*P*^2^
Energy, MJ/d	9.5 ± 0.5	9.4 ± 0.5	−0.09 ± 0.4	9.6 ± 0.5	9.2 ± 0.4	−0.46 ± 0.3	9.2 ± 0.5	9.7 ± 0.5	0.50 ± 0.4	0.149
Total fat, % of TE	34.6 ± 1.0	32.2 ± 1.2	−2.4 ± 0.8	34.8 ± 1.2	31.8 ± 1.1	−3.0 ± 0.9	35.2 ± 1.2	32.3 ± 1.2	−2.9 ± 1.1	0.906
SFA, % of TE	12.9 ± 0.6	12.7 ± 0.8	−0.2 ± 0.6	12.4 ± 0.7	11.7 ± 0.7	−0.7 ± 0.4	13.4 ± 0.6	12.2 ± 0.7	−1.2 ± 0.6	0.391
MUFA, % of TE	11.7 ± 0.5	10.8 ± 0.4	−1.0 ± 0.3	12.0 ± 0.5	10.9 ± 0.4	−1.1 ± 0.4	12.3 ± 0.6	11.0 ± 0.5	−1.3 ± 0.5	0.981
n–6 PUFA, % of TE	4.81 ± 0.2	4.11 ± 0.2	−0.70 ± 0.2	5.22 ± 0.4	4.23 ± 0.2	−0.99 ± 0.3	4.58 ± 0.3	4.37 ± 0.3	−0.21 ± 0.3	0.464
n–3 PUFA, % of TE	0.78 ± 0.1	0.79 ± 0.1	0.01 ± 0.6	0.84 ± 0.1	0.85 ± 0.1	0.01 ± 0.6	0.74 ± 0.1	0.71 ± 0.1	−0.03 ± 0.6	0.495
Total PUFA, % of TE	5.59 ± 0.3	5.04 ± 0.3	−0.55 ± 0.4	6.06 ± 0.4	4.81 ± 0.3	−1.24 ± 0.4	5.32 ± 0.3	5.08 ± 0.3	−0.24 ± 0.4	0.456
*trans* Fat, % of TE	0.88 ± 0.1	0.90 ± 0.1	0.02 ± 0.1	0.97 ± 0.1	0.95 ± 0.1	−0.02 ± 0.1	1.00 ± 0.1	0.97 ± 0.1	−0.03 ± 0.1	0.972
Protein, % of TE	16.0 ± 0.5	24.4 ± 0.9	8.4 ± 0.8^a^	15.9 ± 0.5	24.4 ± 0.7	8.5 ± 0.6^a^	16.2 ± 0.6	15.2 ± 0.6	−1.00 ± 0.6^b^	<0.001
Carbohydrates, % of TE	49.4 ± 1.3	44.0 ± 1.2	−5.5 ± 1.0^a^	49.7 ± 1.4	43.7 ± 1.4	−6.0 ± 1.3^a^	48.5 ± 1.7	54.8 ± 1.6	6.3 ± 1.9^b^	<0.001
Alcohol, % of TE	3.02 ± 0.7	2.60 ± 0.5	−0.42 ± 0.5	2.59 ± 0.6	2.76 ± 0.6	0.18 ± 0.4	3.20 ± 0.7	2.48 ± 0.7	−0.72 ± 0.6	0.066
Dietary fiber (AOAC), g/d	22.6 ± 1.4	20.1 ± 1.2	−2.49 ± 1.1	23.0 ± 1.4	20.5 ± 1.2	−2.56 ± 0.8	21.3 ± 1.1	20.4 ± 1.3	−0.92 ± 0.9	0.399
Sodium, g/d	3.18 ± 0.2	3.12 ± 0.2	−0.07 ± 0.2	3.37 ± 0.2	2.81 ± 0.2	−0.57 ± 0.2	3.57 ± 0.5	2.95 ± 0.2	−0.62 ± 0.5	0.215
Potassium, g/d	5.22 ± 1.2	5.21 ± 1.2	−0.01 ± 0.2	4.54 ± 0.7	4.71 ± 0.9	0.17 ± 0.4	4.10 ± 0.6	4.80 ± 0.9	0.70 ± 0.8	0.582
Calcium, g/d	1.06 ± 0.1	1.19 ± 0.1	0.13 ± 0.1^b^	1.09 ± 0.1	1.57 ± 0.1	0.48 ± 0.1^a^	0.97 ± 0.1	1.00 ± 0.1	0.03 ± 0.1^b^	<0.001

1 All values are means ± SEMs. *n* = 38. Data were log transformed for alcohol intake. Baselines were not significantly different (*P* ≤ 0.05) from one another. Different superscript letters within a row refer to treatment groups different from one another (*P* ≤ 0.05). AOAC, Association of Official Analytic Chemists; TE, total energy; Δ change from baseline.

2 Overall between-group treatment effects for each Δ were obtained with the use of a linear mixed-model ANOVA with baseline values for the variable of interest and prognostic values such as age, sex, and BMI. Tukey-Kramer correction was used for the post hoc analysis to adjust for multiple testing.

### ACEi effects of study drinks: in vitro study

ACEi effects of the intervention products are presented in **Supplemental Figure 2**. Whey-protein permeate exhibited significant ACEi activity compared with that of calcium-caseinate permeate, control permeate and of undigested gastric digesta and retentate for whey protein, calcium caseinate, and the control.

## DISCUSSION

This novel study revealed that the consumption of whey protein (56 g/d) for 8 wk resulted in clinically relevant reductions in 24-h SBP (−2.9 ± 1.1 mm Hg) and DBP (−2.0 ± 0.7 mm Hg) compared with the effect of the control in adults with prehypertension and mild hypertension. To date, only a very limited number of studies have evaluated the effects of intact dairy proteins on BP. Although Pal and Ellis (7) did not use 24-h AMBP, our findings support their data relating to the efficacy of whey protein to reduce BP. However, contrary to their findings, we did not detect changes in BP after calcium-caseinate supplementation. Although clinic BP may be considered less reliable in relation to future cardiovascular mortality and morbidity (30, 31), we also showed a significant decrease in clinic SBP after whey-protein supplementation than after control intake. The inhibition of ACE has been proposed as a potential mechanism by which dairy proteins reduce BP (32). Data from our in vitro study confirmed the significant ACEi activity of whey protein compared with that of calcium caseinate and the control. However, a reduction in circulating ACE activity was not observed in the intervention study, possibly because of a lack of power, and further studies are require to confirm the importance of ACEi in BP reduction by whey protein.

Significant improvements in the %FMD response after both whey protein and calcium-caseinate supplementation compared with after control intake were also observed, thereby supporting previous studies (33, 34). FMD is the gold standard for endothelial dysfunction assessment and provides additional, independent prognostic data beyond the classic CVD-risk factors (35). It has been estimated that the clinical significance of consuming whey protein and calcium caseinate for 8 wk would be 7.7% and 1.4% reductions in future CVD events, respectively (36). Nitric oxide has been recognized as a mediator of vessel dilation (37). However, we observed no differences in plasma NO_2_ or NO_3_ between intervention products, thereby confirming previous data (33). Nevertheless, it has been proposed that vascular dilation is not solely related to nitric oxide (38). Indeed, inflammatory markers and cell adhesion molecules have also been associated with the progression of atherosclerosis and endothelial dysfunction (39). During atherosclerotic plaque formation, inflammatory cells, circulatory soluble adhesion molecules (such as sICAM-1 and sVCAM-1), and cell surface adhesion molecules (E-selectin and P-selectin) are activated (40). We showed a significant decrease in sVCAM-1 and sICAM-1 after calcium-caseinate and whey-protein consumption, respectively, than after control intake. This result suggests that the beneficial effects of dairy proteins on adhesion molecules may be a potential mechanism for improvements in vascular reactivity. This outcome is in agreement with a recent review that summarized the acute and chronic effects of dietary proteins on markers of inflammation and adhesion molecules and reported a reduction in sICAM after a 4-wk high-protein diet compared with the effect of maltodextrin (41).

In contrast with our study, Pal and Ellis (7) reported a decrease in the augmentation index after whey-protein supplementation. Nevertheless, we showed significant decreases in systolic and mean arterial pressures of both peripheral and central pressures that were assessed with the use of a pulse-wave analysis. This result is an important finding because emerging evidence suggests that aortic pressure is more strongly related to future cardiovascular events than is brachial pressure (42–44) because organs such as the heart, brain, and kidneys are exposed to central pressure rather than peripheral pressure (45). Furthermore, the mean arterial pressure has been proposed to be a predictor of stroke (46). Note that, in our study, each intervention period lasted for 8 wk as opposed to in the study of Pal and Ellis (7), which was a 12-wk intervention. At week 6, Pal and Ellis (7) showed no significant treatment effects on the augmentation index, which suggests that a longer intervention period may have been required for significant changes to the augmentation index to be shown. This possibility has been supported by Jauhiainen et al. (47) who reported a lower augmentation index after lactotripeptide consumption in a 12-wk trial. In contrast, improvements in the pulse-wave velocity (which is another measure of arterial stiffness) after 6 and 8 wk of supplementation with lactotripeptides were observed (48, 49).

A significant decrease in total cholesterol after both whey protein and calcium-caseinate supplementation was observed as were reductions in triacylglycerol after whey-protein consumption. In our recent review, we identified 4 RCTs and a pilot study that evaluated the long-term effects of dairy proteins on lipid metabolism (50). Our results are in agreement with the majority of these studies (51–53); however, we failed to detect a decrease in LDL cholesterol or an increase in HDL cholesterol. Longer supplementation periods with milk proteins may have been more informative on the relative effects of dairy proteins on other lipid profiles or on the strength of significance. Calcium intake from dairy has been associated with calcium–fatty acid soap formation in the gastrointestinal tract, which leads to reduced fat absorption (54, 55); thus, calcium intake may well be a mechanism of action for the lipid-lowering effects of calcium caseinate. Although calcium has been linked with a favorable effect on BP (56), calcium-caseinate supplementation had no significant effect on BP despite containing >2.5-fold higher calcium than whey protein. Other potential mechanisms of action have been linked with both whey protein and calcium-caseinate consumption. These mechanisms include the inhibition of genes that are involved in fatty-acid and cholesterol absorption and synthesis in the intestines (57, 58), increased catabolic metabolism via urinary excretion of tricarboxylic acid cycle compounds (59), and the stimulation of gut-bacteria activity resulting in higher short-chain fatty acids (60), which have been proposed as key regulatory metabolites in lipid metabolism (61).

There was no change in body weight or dietary intake apart from the inclusion of study products during the intervention. Isoenergetic intake was successfully achieved through diligent dietetic management, which increased the motivation and compliance of our participants. The habitual milk and dairy consumption of the participants was similar to that of the United Kingdom population (62) and confirmed that the favorable effects of dairy proteins were observed in individuals who habitually consume dairy foods.

To blind the study, maltodextrin was chosen as an appropriate control because it was flavorless and easily dissolved and had an energy content that matched that of the milk proteins. However, the maltodextrin may not have been inert and could have influenced some of the variables measure, which could have been a potential limitation of the study. Nevertheless, the differences observed between the whey protein and calcium-caseinate groups confirmed the effects attributed to the specific properties of the proteins rather than to the differences between macronutrients. Another potential limitation was that the daily dose given in this trial was relatively high, and therefore further studies are required to determine the lowest effective dose of dairy proteins.

In conclusion, this novel RCT has several important observations. Compared with the control, whey protein significantly lowered 24-h SBP and DBP, central and peripheral SBP, and mean arterial pressure. Furthermore, compared with the control, both whey protein and calcium caseinate improved endothelial function, reduce adhesion molecules and vascular biomarkers of risk, and improved blood lipids. The magnitude of changes in the CVD risk markers observed is modest but may have important implications for public health.

## Footnotes

6 Abbreviations used: ACE, angiotensin-converting enzyme; ACEi, angiotensin-converting enzyme inhibitory; AMBP ambulatory blood pressure; BP, blood pressure; CVD, cardiovascular disease; DBP, diastolic blood pressure; DVP, digital volume pulse; FFQ, food-frequency questionnaire; FMD, flow-mediated dilatation; RCT, randomized controlled trial; SBP, systolic blood pressure; sICAM-1, soluble intercellular adhesion molecule 1; sVCAM-1, soluble vascular cell adhesion molecule 1; %FMD, flow-mediated dilatation percentage.
